# Generative Adversarial Network Technologies and Applications in Computer Vision

**DOI:** 10.1155/2020/1459107

**Published:** 2020-08-01

**Authors:** Lianchao Jin, Fuxiao Tan, Shengming Jiang

**Affiliations:** College of Information Engineering, Shanghai Maritime University, Shanghai 201306, China

## Abstract

Computer vision is one of the hottest research fields in deep learning. The emergence of generative adversarial networks (GANs) provides a new method and model for computer vision. The idea of GANs using the game training method is superior to traditional machine learning algorithms in terms of feature learning and image generation. GANs are widely used not only in image generation and style transfer but also in the text, voice, video processing, and other fields. However, there are still some problems with GANs, such as model collapse and uncontrollable training. This paper deeply reviews the theoretical basis of GANs and surveys some recently developed GAN models, in comparison with traditional GAN models. The applications of GANs in computer vision include data enhancement, domain transfer, high-quality sample generation, and image restoration. The latest research progress of GANs in artificial intelligence (AI) based security attack and defense is introduced. The future development of GANs in computer vision is also discussed at the end of the paper with possible applications of AI in computer vision.

## 1. Introduction

Computer vision (CV) is a science that studies how to make a machine “see.” In 1963, Larry Roberts from MIT published the first doctoral dissertation in this field, “Machine Perception of Three-Dimensional Solids”, marking the beginning of CV research as a new direction of artificial intelligence. The background of this field was initially inspired by the human visual system, which is divided into two parts: the brain and eye. They cooperate so that the human visual system can easily explain any scene. For example, it can distinguish among tens of thousands of categories of objects through learning in the process of people's growth and can find specific goals in a very short time in a particular scene. It is easy to switch between several types of recognition processes with flexibility and rapidity. However, its complexity and dynamics have not been well understood and explained at present [1]. This field is to create a system with the same perception ability as the human visual system. And this system has been very diverse and complex after decades of development.

Computer vision is one of the most popular research directions in the field of deep learning at present. It is a cross-disciplinary subject, including computer science and technology (e.g., theory, systems, graphics, algorithms, and architecture), advanced mathematics (e.g., information retrieval, machine learning, probability, and statistics), information engineering (e.g., robotics, speech, natural language processing, and image processing), physics (e.g., optics), biology (e.g., neuroscience), and psychology (e.g., cognitive science). Many directions overlap with computer vision, which mainly involves image processing and machine vision. Other fields include pattern recognition, computer graphics, AI medicine, automatic driving, anomaly detection, AI-based attack, and defense. So far, there are many definitions of computer vision; several rigorous ones are summarized as follows from the relevant literature:

1. Computer vision is an explicit and meaningful description of the construction of objective objects in images
2. Computer vision calculates the characteristics of the three-dimensional world from one or more digital images
3. Computer vision is based on perceptual images to make useful decisions for objective objects and scenes

Computer vision is most widely used in image processing. It includes many aspects, such as image classification, image recognition, image detection, image super-resolution, and domain transfer [2–6]. With the rapid development of deep learning schemes, image restoration technology for missing information [7, 8] has also achieved great success in recent years. Due to the insufficient storage of image databases in some fields, such as medical image and underwater image, the generalization ability of models trained with a small amount of data is poor. So in recent years, data enhancement technology [9, 10] has succeeded by generative models such as GANs. The increase of data volume makes the training effect of neural networks better and the generalization ability and robustness of models stronger, achieving higher accuracy in image recognition.

Visual recognition is a key component of computer vision, consisting of three processes: classification, recognition, and detection. The rapid development of neural networks and deep learning has greatly promoted the development of these most advanced visual recognition systems. With the continuous progress of computer memory and computing speed as well as the support of huge information data, a large number of fast-growing and practical applications have emerged in this field. For example, with the rapid development of 5G networks, a computer vision system can collect real-time traffic signs, lights, pedestrians, and vehicles on the road and transmit them to the host computer for real-time control of vehicles to achieve automatic driving [11]. In the field of biometric recognition, computer vision provides many feasible schemes, which make face recognition, face matching, and fingerprint recognition technologies to penetrate all aspects of our lives. For example, the newly operating Beijing Da Xing National Airport has fully realized the inbound and outbound facial recognition. In public security supervision, surveillance cameras used to monitor suspicious behavior are widely distributed in public places. Real-time personnel monitoring is realized through target detection [12, 13] and faces recognition technologies [14–18], which provides effective help for hunting suspicious persons. Microsoft Kinect has successfully developed related game applications using stereo vision technologies in-game and control. Netease Fuxi Laboratory established virtual game characters through GANs [19]. Among the major search websites, Google Images has successfully used content-based query technologies to search related pictures, using algorithms to analyze the content of the query image, returning the results according to the best matching content. In recent years, computer vision has been greatly promoted by the development of deep learning schemes, which have made great progress in the field of data processing [20, 21], accordingly promoting the rapid development and application of computer vision in many fields.

Computer vision is closely related to AI. The mature technologies of computer vision can be applied to AI. Computer vision has a large number of basic applications of quantum AI. Many scientists believe that computer vision has opened the way for the development of AI. However, there are also essential differences between them. AI emphasizes reasoning and decision-making, while computer vision is still mainly for image information expression and object recognition. “Object recognition and scene understanding” also involve image feature reasoning and decision-making. However, it is still different from AI reasoning and decision-making [22]. For example, Alpha Go and Alpha Zero are not considered as computer vision but typical AI contents instead.

In this survey paper, the research progress of GANs is elaborated in detail with the following organization. In the second section, the theoretical basis of GANs is introduced. The challenges and technical advantages of GANs compared are discussed in comparison with traditional machine learning schemes. In the third section, we introduce some new derivative models on loss function and model structure in comparison with the traditional GAN models, along with analyzing the hidden space of GANs. In the fourth section, the latest applications of GANs in computer vision are introduced, including data enhancement, high-quality sample generation, domain transfer, image restoration, and AI security. This paper is concluded in the fifth section by summarizing the development process of GANs and the future development direction of GANs in computer vision.

## 2. Basic Theory of GANs

GANs are generative models proposed by Goodfellow et al. in 2014 [23]. Since the introduction of GANs, many interesting applications in image generation and other fields have arisen, the model has been applied to various tasks of computer vision. Generally speaking, generative models are classified into two categories. The first is the traditional generation algorithm based on machine learning, which consists of Restricted Boltzmann Machine (RBM) [24], Naïve Bayes Model (NBM) [25], and Hidden Markov Model (HMM) [26]. The other is deep learning models consisting of the automatic encoder (VAE) [27], GANs, and its derivative models. GAN is a generative model that generates target data by latent variables. Specifically, game training is conducted between generator and discriminator in the model, and target variables with real data distribution are generated by random variables (usually obeying Gauss distribution). Compared with the traditional machine learning algorithm, the model is simpler and more functional and has more application scenarios. And it has better performance than traditional algorithms on large data sets, such as ImageNet [28] and CIFAR-100.

The development process of GANs can be roughly summarized into three stages. The development process of each stage is shown in Table 1. DCGAN and WGAN are the milestones of each stage. DCGAN represents a significant generative model. Compared with previous GANs, this model becomes more easily controlled and the model is not easy to collapse. The shortcomings and technical advantages of GANs will be described in Sections 2.2 and 2.3. The emergence of WCGAN has brought the GAN models to a new height. This model can generate higher quality samples [29]. According to the requirements of different tasks and different scenarios, different GAN models have been proposed one after another and have been widely used in the field of computer vision. Moreover, it also has a good performance in cross-domain fields, such as medical, art, and security encryption fields. BigGAN [30] first generated images with high fidelity and low variety gap, the accuracy has been improved by leaps and bounds, and it is an important milestone in the development history of GAN. StyleGAN [31] is another breakthrough in the field of GAN research. StyleGAN has created a new record in facial generation tasks. The core of the algorithm is style transfer technology or style mixing. In addition to generating faces, it can also generate high-quality images of cars, bedrooms, etc. In the use cases of face aging and other industries, Age-cGAN [32] is very useful in cross-age face recognition, finding missing children, and entertainment.

Not only that, with the further research of GANs, they not only develop rapidly in image processing and video processing but also can be used in speech processing, text processing, and signal processing fields such as speech super-resolution, text-synthesized images, ECG, and EEG signal recognition. Recent research results show that GANs can be combined with the field of signal communication to achieve combinatorial optimization design [7]. Therefore, GANs and its derivative models have strong vitality, and its research and application are in the ascendant stage, which has a broad application prospect in future development.

### 2.1. GAN Network Structure

**Figure 1:**
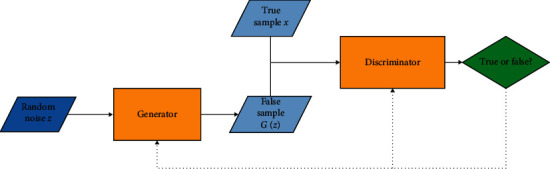
GAN network architecture.

GAN is popular because of its unique network model architecture. GAN consists of two parts: generator and discriminator. Generator and discriminator are usually implemented by neural networks [33]. The inspiration for the GAN model comes from the minimax two-player game. Generators generate samples with approximate real data distribution through random data. Discriminators need to discriminate between true samples and false samples. Game training is used to optimize model weight parameters between the two networks to improve the generalization ability of the model. Finally, the data distribution of false samples generated by the generator is more in line with the data distribution of true samples, while the discriminator has half the probability to distinguish the true samples and half the probability to distinguish the false samples. The ideal state of the model is that the discriminator cannot distinguish the false samples from the true samples eventually to achieve an indivisible equilibrium state [34].  Figure 1 shows the network architecture of GAN. Implicit variable *Z* generates false samples *G* (*z*) by the generator. The discriminator determines the authenticity of two kinds of input data (true samples and false samples), outputs their discrimination probability through objective function, and optimizes the two network structures.

The loss function of GAN is based on the minimax game of two gamers, which includes two neural networks competing with each other in the framework of a zero-sum game [35]. The discriminator needs to distinguish the input data from the true one and optimize the weights of the network model by the backpropagation algorithm. The input parameters of the discriminator are *X* and *θ*^(*D*)^. The loss function of the discriminator is

(1) $$\begin{array}{c} V\left(D,\theta^{\left(D\right)}\right)=-E_{x∼p_{r}\left(x\right)}\left[\log\;D\left(x\right)\right]-E_{z∼p_{g}\left(z\right)}\left[\log\left(1-D\left(G\left(z\right)\right)\right)\right]. \end{array}$$

Among them, *P*_*r*_ represents the data distribution of true samples and *p*_*g*_ represents the data distribution of false samples generated by the generator. The input parameters of the generator are *Z* and *θ*^(*G*)^, and the loss function is as follows:

(2) $$\begin{array}{c} V\left(G,\theta^{\left(G\right)}\right)=E_{z∼p_{g}}\left[-\log\left(D\left(G\left(z\right)\right)\right)\right]. \end{array}$$

The discriminator and the generator optimize the weights *θ*^(*D*)^ and *θ*^(*G*)^ of each model through loss function, respectively. The generator and the discriminator do not change the model parameters of each other when they are trained. GAN will not stop training until the two network structures reach the Nash equilibrium [36]. In the GAN model, two kinds of network structures are trained with the adversarial model. The final objective function of GAN model is as follows:

(3) $$\begin{array}{c} \underset{G}{\min}\underset{D}{\max}V\left(G,D\right)=\underset{G}{\min}\underset{D}{\max}E_{x∼p_{r}\left(x\right)}\left[\log\;D\left(x\right)\right]+E_{z∼p_{g}\left(z\right)}\left[\log\left(1-D\left(G\left(z\right)\right)\right)\right]. \end{array}$$

In GAN training, generators and discriminators are trained alternately. First, discriminator *D* is trained, and then generator *G* is trained. When one of the network structures is trained, the other structure is fixed, so alternating training two networks one by one. In theory, to get the optimal solution for *V* (*D*, *G*), discriminator should be trained *K* times firstly and then the generator should be trained once. But in practice, that *K* is equal to 1 is found to be more suitable.

*V* (*G*, *D*) is a common cross-entropy loss problem in binary classification. From the formula, it is a binary classification problem for discriminator *D*. To ensure that the *V* (*G*, *D*) can achieve the maximum value, the following *D*^*∗*^(*x*) can be obtained after derivation of *V* (*G*, *D*):

(4) $$\begin{array}{c} D^{*}\left(x\right)=\frac{p_{r}\left(x\right)}{p_{r}\left(x\right)+p_{g}\left(x\right)}. \end{array}$$

The *KL* divergence of *P*_*r*_(*x*) and *p*_*g*_(*x*) can be obtained by introducing the above formula into the objective function, which can better explain the training of the model. The specific explanation can be found in Section 2.3. For generator *D*, since there is no log *D*(*x*) item in the model, so the generator's objective function can be simplified as follows:

(5) $$\begin{array}{c} \underset{G}{\min}V\left(G,D\right)=\underset{G}{\min}E_{z∼p_{g}\left(z\right)}\left[\log\left(1-D\left(G\left(z\right)\right)\right)\right]. \end{array}$$

For the whole GAN network, the central idea is attributed to the Nash equilibrium of game theory [37]. The model needs to train discriminators to improve the discriminant rate between true samples and false samples. At the same time, generators need to constantly minimize log (1 − *D* (*G* (*z*)) to generate more realistic samples to confuse the discriminator. GAN network trains the model by alternating optimization method, when and only when *p*_*r*_ = *p*_*g*_, and GAN reaches the global optimal solution.

### 2.2. Challenges of GANs

Although GANs have achieved good results in many fields, GANs also face some challenges, such as the gradient disappearance in training, the phenomenon of model collapse in unstable training, the poor diversity of GAN generators, and uncontrollable training.

The main problem of GAN is to minimize and measure the distance between the two distributions *p*_*r*_ and *p*_*g*_. When the generator is fixed, the training of discriminator is also a process of minimizing the cross-entropy.

In the GAN network structure introduced in Section 2.1, from (4) and (3), we can obtain the following formula:

(6) $$\begin{array}{c} V\left(D,\theta^{\left(G\right)}\right)=\text{KL}\left(\left.p_{r}\right\|\frac{p_{r}+p_{g}}{2}\right)+\text{KL}\left(\left.P_{g}\right\|\frac{p_{r}+p_{g}}{2}\right)-2\;\log\;2. \end{array}$$

KL represents KL divergence; KL is an asymmetric measure of similarity between two distributions. Its definition is shown in

(7) $$\begin{array}{c} \text{KL}\left(\left.p_{r}\right\|p_{g}\right)=E_{x∼p_{r}}\left[\log\frac{p_{r}\left(x\right)}{p_{g}\left(x\right)}\right]. \end{array}$$

Equation (8) is another JS divergence that defines distance:

(8) $$\begin{array}{c} \text{JSD}\left(\left.p_{r}\right\|p_{g}\right)=\frac{1}{2}\text{KL}\left(\left.P_{r}\right\|\frac{p_{r}+p_{g}}{2}\right)+\frac{1}{2}\text{KL}\left(\left.P_{g}\right\|\frac{p_{r}+p_{g}}{2}\right). \end{array}$$

Through (7) and (8), (6) can be changed as follows:

(9) $$\begin{array}{c} V\left(D,\theta^{\left(G\right)}\right)=2\text{JSD}\left(\left.p_{r}\right\|p_{g}\right)-2\;\log\;2. \end{array}$$

Therefore, for optimizing the objective function of GAN, the ultimate goal is to optimize the *JS* divergence of generator and discriminator, making the distribution of *p*_*g*_ and *p*_*r*_ more similar. When the two distributions do not intersect, their *JS* divergence is a constant −2 log 2. At this time, the gradient of the generator and discriminator is 0, which makes model training more difficult. Because the parameter of generator input data is low dimension and the parameter dimension of the real sample is generally high, so the overlap area of *p*_*g*_ and *p*_*r*_ is too less to calculate, which leads to the gradient disappearance.

The original GAN generator has two loss functions, one loss function shown in (2) and the other loss function as follows:

(10) $$\begin{array}{c} V\left(G\right)=E_{x∼p_{g}}\left[-\log\;D\left(x\right)\right]. \end{array}$$

We can transform the KL divergence into the form with *D*^*∗*^:

(11) $$\begin{array}{c} \text{KL}\left(\left.p_{g}\right\|p_{r}\right)=E_{x∼p_{g}}\left[\log\frac{p_{g}\left(x\right)}{p_{r}\left(x\right)}\right]\\ =E_{x∼p_{g}}\left[\log\frac{p_{g}\left(x\right)/\left(p_{r}\left(x\right)+p_{g}\left(x\right)\right)}{p_{r}\left(x\right)/\left(p_{r}\left(x\right)+p_{g}\left(x\right)\right)}\right]\\ =E_{x∼p_{g}}\left[\log\frac{1-D^{*}\left(x\right)}{D^{*}\left(x\right)}\right]\\ =E_{x∼p_{g}}\log\left[1-D^{*}\left(x\right)\right]-E_{x∼p_{g}}\log D^{*}\left(x\right). \end{array}$$

From (9)‒(11), we can get the equivalent deformation of the minimum target:

(12) $$\begin{array}{c} E_{x∼p_{g}}\left[-\log D^{*}\left(x\right)\right]=\text{KL}\left(\left.p_{g}\right\|p_{r}\right)-E_{x∼p_{g}}\log\left[1-D^{*}\left(x\right)\right]\\ =\text{KL}\left(\left.p_{g}\right\|p_{r}\right)-2\text{JS}\left(\left.p_{r}\right\|p_{g}\right)+2\log2+E_{x∼p_{r}}\left[\log D^{*}\left(x\right)\right]. \end{array}$$

The last two terms of the above formula do not depend on *G*. The minimization (10) is equivalent to minimization:

(13) $$\begin{array}{c} \text{KL}\left(\left.p_{g}\right\|p_{r}\right)-2\text{JS}\left(\left.p_{r}\right\|p_{g}\right). \end{array}$$

There are two problems in (13). Firstly, the KL divergence of generated distribution and real distribution should be minimized, while the JS divergence of both should be maximized. Secondly, because KL divergence is asymmetrical, KL(*p*_*g*_‖*p*_*r*_) is different from KL(*p*_*r*_‖*p*_*g*_).

When

(14) $$\begin{array}{c} p_{g}\left(x\right)⟶0,p_{r}\left(x\right)⟶1:p_{g}\left(x\right)\log\frac{p_{g}\left(x\right)}{p_{r}\left(x\right)}⟶0,\text{KL}\left(\left.p_{g}\right\|p_{r}\right)⟶0, \end{array}$$

the model lacks diversity; when

(15) $$\begin{array}{c} p_{g}\left(x\right)⟶1p_{r}\left(x\right)⟶0:p_{g}\left(x\right)\log\frac{p_{g}\left(x\right)}{p_{r}\left(x\right)}⟶\infty ,\text{KL}\left(\left.p_{g}\right\|p_{r}\right)⟶\infty , \end{array}$$

the model lacks accuracy and the generator cannot find a balance between them, so it is easy to cause model collapse.

In the original loss function (2), we face the problems of gradient disappearance and training difficulties. When (10) became the loss function of the generator, it will face the problems of optimization objective uncertainty, gradient instability, and model collapse [38]. So, the main problem of GAN is to choose the right distance function at present. We need to find a distance function that is better than JS divergence or KL divergence to make the model optimization more reasonable, facilitate the model convergence, and make the distribution *p*_*g*_ more similar to *p*_*r*_ [7].

### 2.3. Advantages of GANs

Compared with other generative models, GANs have two characteristics. First, GANs do not rely on any prior assumptions. Many traditional methods assume that the data obey a certain distribution and then use maximum likelihood to estimate the data distribution. GAN training is simpler and more diverse. Secondly, the way to generate real-like samples is very simple. GAN generates real-like samples by forward propagation of generator to generate deceptive data, while the traditional sampling method is very complex. The emergence of GAN overturns the traditional artificial intelligence algorithm which restricts people's thinking. It provides a powerful method for unsupervised deep learning models. GAN uses machines to interact with machines through continuous adversarial training. After sufficient data training, it can learn the inherent laws in the real world.

## 3. Evolution of GANs

With the continuous exploration of the GAN field, more and more GAN derivative models emerge in an endless stream. To deal with different problems, different GAN derivative models play a great role in their respective fields. Aiming at the problems and challenges faced by GAN described in Section 2, the problems in GAN models can be roughly divided into two categories: one is to improve the loss function in dealing with gradient disappearance and model collapse in the derivative models of GAN; the other is to improve the model structure of GAN for the problems of poor sample richness. In the next two sections, this paper will elaborate on the latest development and exploration of the GAN model evolution in the different domains.

### 3.1. Loss Function

The selection of loss function is the most important problem for the GAN model. The loss function has a direct influence on the gradient disappearance and the model collapse. For generators, the input data are usually low-dimensional random vectors, and the dimension of real samples is generally high. Generators usually generate high-dimensional false samples through U-net and other neural networks. Because of the difference in dimension space, it may lead to the overlap area of the distribution of true samples and false samples to be 0. The JS divergence measures the distance between the true sample distribution and the false sample distribution will be constant. The gradient disappearance will happen and the training of the model will become very difficult. To achieve the stability of training and reduce the phenomenon of gradient disappearance, different kinds of GAN derivative models based on loss function improvement emerge endlessly.  Table 2 lists the derivative models improved by loss function in recent years while Table 3 shows some comparisons on four main models. For each improved model, the discriminator loss function and generator loss function are given, respectively. StackGAN is a two-stage generative model. The discriminator loss function and generator loss function of the StackGAN++ model improved by the StackGAN model are given in Table 2. The table only lists the improved GAN model based on loss function, which is tabular in recent years. It can be seen that the change of loss function is a popular trend for the improvement of the GAN model.

WGAN uses Wasserstein distance instead of JS divergence to solve gradient disappearance and unstable model training in vanilla GAN. The Wasserstein distance is

(16) $$\begin{array}{c} W\left(p_{r},p_{g}\right)=\underset{\gamma ∼\prod \left(p_{r},p_{g}\right)}{\inf}E_{\left(x,y\right)}\left[\left\|x-y\right\|\right]. \end{array}$$

∏(*p*_*r*_, *p*_*g*_) is a set of all possible joint distributions combined by *P*_*r*_ and *P*_*g*_. For each possible joint distribution *γ*, we can get a real sample *x* and a generated sample *y* from the sample (*x*, *y*) ~ *γ* and calculate the distance ‖*x* − *y*‖ of the pair of samples. In all possible joint distributions, the lower bound of the expected value is defined as Wasserstein distance.

The advantage of Wasserstein distance compared with KL divergence and JS divergence is that even if the two distributions do not overlap, Wasserstein distance can still reflect their distance. Because of its superior smoothing characteristics compared with KL divergence and JS divergence, it can theoretically solve the problem of gradient disappearance. Then, the Wasserstein distance is written as a solvable form by mathematical transformation, and the Wasserstein distance can be approximated by maximizing this form by using a discriminator neural network with a limited parameter value range. Under this approximate optimal discriminator, the distance of Wasserstein is reduced by optimizing the generator, which can effectively narrow the generated distribution and the real distribution. WGAN not only solves the problem of unstable training but also provides a reliable index of the training process, which is highly related to the quality of generated samples.

**Figure 2:**
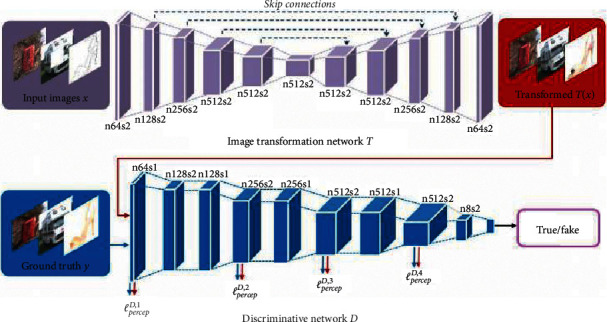
PAN network structure [40].

PAN [40] is a perceptual adversarial network improved by the loss function. In the training of the model, the difference between the true sample and the false sample is constantly found by discriminator and the weight parameters of the network are updated by calculating the value of the loss function to improve the accuracy of sample recognition. The generator also updates the parameters of the network by calculating the value of the loss function to generate more realistic false samples. PAN adds a loss function called the perceptual adversarial loss to the traditional GAN loss function. The loss function calculates the difference between the eigenvalues of true samples and false samples extracted from different layers of the discriminator network when discriminating between true samples and false samples in cyclic training. The difference is added to the loss function of the traditional generator and discriminator. The respective objective functions are shown in Table 2, and the calculation of the difference between the true samples and the false samples in distinguishing the high-level features of the discriminator network is shown in (11). PAN network is superior to traditional GAN network in image-to-image conversions, such as image dewatering and image restoration. Its network structure is shown in Figure 2:

(17) $$\begin{array}{c} \ell_{\text{percep}}^{D,j}=\frac{1}{n}\sum_{i=1}^{n}\left\|d_{j}\left(y_{i}\right)-d_{j}\left(G\left(x_{i}\right)\right)\right\|. \end{array}$$

Building footprint information is an important part of the three-dimensional reconstruction of the urban model. The automatic generation of building footprint images from satellite images is a considerable challenge. To improve the quality of building footprint image generation, Shi et al. [44] proposed CWGAN based on CGAN and WGAN. There are some problems with the original GAN model. To solve the problems of uncontrollable GAN training and gradient disappearance, Mirza et al. proposed a GAN derivative model with conditional constraints-CGANs. The objective function is shown as follows:

(18) $$\begin{array}{c} L_{\text{CGAN}}=E_{px}\left[\log D\left(\left.x\right|y\right)\right]+E_{pz}\left[\log\left(1-D\left(G\left(\left.z\right|y\right)\right)\right)\right]. \end{array}$$

To solve the problem that the original GAN may collapse during training, Arjovsky et al. proposed an alternative loss function based on Wasserstein distance [39]. The loss function will provide more useful gradient information to the generative network, which will greatly reduce the phenomenon of gradient disappearance and improve the stability of the training model. A new GAN derivative model is thus created-WGAN. CWGAN is based on these two advantages networks. Its objective function is as follows:

(19) $$\begin{array}{c} L_{\text{CWGAN}}=E_{px}\left[D\left(\left.x\right|y\right)\right]-E_{pz}\left[D\left(G\left(\left.z\right|y\right)\right)\right]. \end{array}$$

The experimental results show that the proposed method can significantly improve the performance of the system. Compared with conditional generative adversarial networks, U-net, and other networks, the quality of building footprint generation is improved. Other networks that improve the model collapse by improving the objective function, such as UnrolledGAN [48], have improved the optimization method of generator parameters to solve the problem of the unstable and easy collapse of model training. By expanding and optimizing the discriminator, the generator's objective function is updated dynamically; the diversity and coverage of data are increased.

### 3.2. Model Structure

The improvement of GAN model structure has a good effect on reducing model collapse, improving the stability of model training, and the quality of sample generation, for example, a series of GAN derivative models such as MRGAN, MAD-GAN [49], CGAN, InfoGAN, ACGAN, AdaBalGAN, stackGAN [50], and stackGAN++. This is improved on the structure of the traditional GAN models, which make the quality and diversity of generated samples better than the traditional GAN model.  Table 4 shows comparisons on some main GAN models on the structure.

**Figure 3:**
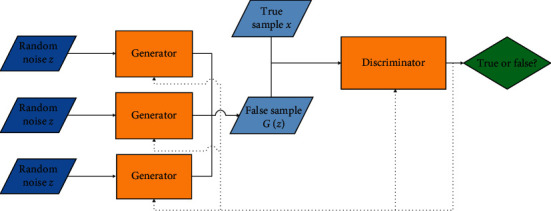
MAD-GAN model structure.

MAD-GAN (Multiagent diverse GAN model) uses multiple generators and a discriminator to solve the problem of poor sample diversity generated by one generator. The model structure is shown in Figure 3. Compared with the traditional GAN, two generators are added, so a regular term is added to the loss function design, which uses cosine distance to punish the consistency of the samples generated by the three generators. MRGAN improves the stability of the model by adding another discriminator, which is used to punish the samples generated when the model training approaches collapse. The model has three loss functions during training. Each function is used to guide the generation of false samples, and the additional discriminator can also judge whether the generated samples are diverse, thus avoiding the phenomenon of model collapse happen.

**Figure 4:**
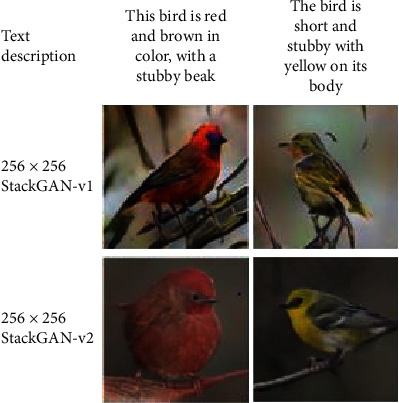
Comparison of stackGAN with its improved version [50] (StackGAN-v1 is original stackGAN while StackGAN-v2 is the stackGAN++ model; the latter can generate more realistic and details samples).

The traditional GAN model is trained alternately by a generator and a discriminator to achieve a Nash equilibrium state. Zhang Han et al. proposed stackGAN and stackGAN++ models, which broke the traditional one-stage training mode and introduced a two-stage training mode. StackGAN is a GAN-derived model for text-generated images. The first stage of this model is to give a text description, such as “This bird has a gray chest and a very short beak.” Through this text description, the shape and color of the original image are outlined from the first stage of the generation model; therefore the low-resolution image is obtained. The second stage generates high-resolution images with real details through the results and text description of the first stage. Recently, the StackGAN++ model has been improved on its original basis, which can execute sample generation under conditional or unconditional generation requirements. The improved model is similar to the spanning tree structure, which contains multiple generators and discriminators. Different branches of the spanning tree can generate different sizes of images in the same scene. Moreover, stackGAN++ has a more stable model structure than stackGAN, and the generated samples are more authentic. The results of the two models are compared as shown in Figure 4. StackGAN and stackGAN++ use this hierarchical structure to solve the problem of the poor effect of GAN in high-resolution image generation. Similar GAN models include GoGAN [51] use a one-stage GAN model, and Progressive GAN [52] generates samples through multiple stages.

### 3.3. Hidden Space Decomposition

In the traditional GAN model structure, implicit variable *Z* (usually random noise with Gauss distribution and text description in text-generated images, etc.) generates false samples through generators. So far, however, it has not been well explained what attributes are controlled by each digit in the implicit variable. Taking the text-generated image as an example, the text description *t* is compiled into a text embedding vector *φ*_*t*_ by the compiler. In the original text-generated image [53, 54], the text is embedded into the nonlinear transformation, and the conditional implicit variable is generated as the input of the generator. Hidden space is generally high-dimensional, but the input is usually low-dimensional; a small amount of data will lead to the discontinuity of implicit variables; for generators, it is not an ideal state. The stackGAN mentioned above generates more conditional variables through conditional extension technology. Specifically, implicit variables are obtained from independent Gauss distribution *N*(*μ*(*φ*_*t*_), *E*(*φ*_*t*_)), and *μ*(*φ*_*t*_) representing the mean of text embedding vectors and *E*(*φ*_*t*_) representing the covariance diagonal matrix of text embedding. Through conditional expansion technology, the diversity and continuity of implicit variables are improved, so more training data will be generated with fewer texts, which makes the model training more stable, and the generalization ability of the model will be greatly improved.

In other derivative models, to improve the stability of model training, implicit variables can be decomposed into a conditional variable *C* and a standard input implicit variable *Z*. The training process can be divided into supervised and unsupervised methods. The supervised methods are mainly composed of CGAN and ACGAN [4], etc. In the supervised training process, implicit variables *Z* and category label *C* are used as input of the generator. In CGAN, the discriminator uses real samples, false samples, and category labels as input to learning the correlation between labels and samples. The discrimination process in ACGAN is the same as traditional GAN, but it will regress the class labels of the samples to learn the correlation between the labels and the samples. The typical unsupervised model is InfoGAN. In the training process, implicit variable *Z* and conditional variable *C* sampled in uniform distribution are used as input of the generator. Mutual information *I*(*c*; *G*(*z*, *c*)) [55] indicates the amount of information about *G* (*z*, *c*) in *C*. The purpose of the discriminator is to enhance the correlation between *C* and the generated results and maximize mutual information. SS-InfoGAN uses a semisupervised approach, dividing the conditional variable *C* into two parts, *c*=*c*_ss_∩*c*_us_: *c*_ss_ is similar to CGAN learning, and *c*_us_ is similar to InfoGAN learning [20].

## 4. Application of GANs in Computer Vision

GAN is one of the most important research directions in the field of computer vision. In particular, in image generation, the image generated by GAN simulation almost reaches the same level as the real picture. Moreover, by changing the network structure of GANs, the generation model can be applied to other fields, such as generative models to generate game characters or game checkpoints, directly generating virtual portraits in the game through portraits. GANs can not only generate images but also play a great role in the text, voice, signal processing, AI security, medical fields, etc.

### 4.1. Data Enhancement

In some areas, the data set reserves are very small, such as the medical image field [56], the artistic and cultural image field [57], and the biological signal field [58]. This will lead to the inadequate training of small data sets, which will lead to the poor generalization ability of the model when using deep learning algorithms. Traditional computer vision image data enhancement technologies include translation, rotation, flip, and scaling, but the diversity and richness of the data obtained by this method are poor. With the continuous development of GANs in computer vision's field, using GAN models to enhance data sets has been applied in many fields.

**Figure 5:**
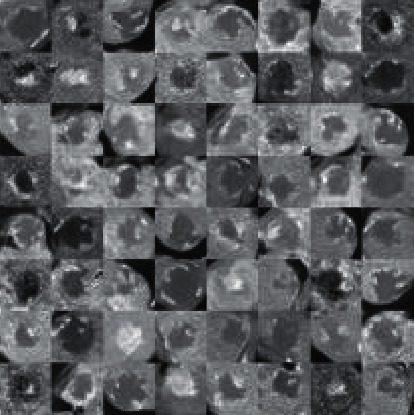
Image of hemangioma generated by CGAN [56].

In the field of medical image, the biggest problem when using a supervised learning machine model to classify images is small data sets and a small number of label samples. The small data sets will lead to inadequate training models. In the past, in the process of collecting medical image data, accurate labeling of images requires a lot of manpower and time. In some data sets exposed in the field of medical images, the data is only the certain specific fields' images, for example, the EEG and ECG. Therefore, too small a data set is the biggest problem in training. NVIDIA researchers used GAN networks to increase the data sets of brain medical CT images with different diseases and showed that the classification performance using only classical data was 78.6% sensitivity and 88.4% specificity. Using data sets enhanced by adding synthetic data, the model could increase to 85.7% sensitivity and 92.4% specificity.  Figure 5 shows an image of the hemangioma synthesized by the above researchers through CGAN.

Biological signals, such as EEG or ECG, can effectively reflect the health status of the human body. The abnormality of biological signals will reflect some symptoms of human diseases. Through the classification of biological signals, we can understand the specific types of diseases reflected. With the development of deep learning models, neural networks perform better than traditional classification models in classification. However, due to the lack of data sets, the accuracy of the classification model is not very high. Haradal Shota et al. [59] proposed a synthesis method of time series data based on GAN and applied GAN models to biological signals synthesis. Specifically, the generator generates synthetic data, the discriminator judges whether the synthetic data is real data, and the generating network and the discriminating network are based on the long short-term memory network (LSTM). The LSTM network is beneficial to generate biological signal time series. In the experiment process, the real data is based on the biological signals: ECG and EEG, and the original dataset is extended by the generative adversarial model. The expansion of original data sets will make the classification model trained more effectively and improve the accuracy of classification. The expansion of data sets thus will help doctors improve the accuracy of diagnosis and determine the direction of treatment.

Besides, Wen et al. applied a neural network to edge calculation and established a data enhancement calculation model. The background of this data model is with the vigorous development of art design, but the application of national cultural elements in researchers is still limited to a few samples and characteristics, and the current artistic design ideas of cultural elements are unclear, have lack of innovation, and have low practical value, which is not conducive to the development of national element art design. To combine national elements with an artistic design closely and promote the healthy development of artistic design, it is necessary to provide sufficient data samples for researchers. Given the above background, Wen and others generated a large number of samples with artistic elements through the GAN model. The data enhancement model solves the problems of low resolution, single feature, and too little data quantity. Moreover, GAN is used to generate innovative images, enrich the elements of national culture, and provide more samples of artistic elements for researchers on national culture study.

The ultimate goal of data enhancement is to increase the data set and improve the training effect of the model. Whether the data generated by GAN can achieve the same training effect as the real data, it is necessary to evaluate the generated data. The first method is to train the model only with false data and test the accuracy of model classification with real data. The second method is the model trained with real data to test whether the generated data is reasonable. Through the above methods, the data set can be expanded with the generated data to improve the model generalization ability.

### 4.2. High-Quality Sample Generation

In the field of computer vision, GANs are widely used as generating models. The ability to learn the distribution of real samples through GANs can not only generate higher resolution images [15, 60] but also play an increasingly important role in the fields of video super-resolution [61], speech super-resolution [14], image enhancement [62], etc. GAN works in an end-to-end manner; it learns the feature distribution and mapping relationship of real samples better than traditional machine learning algorithms. With the wide application of GANs, more and more derivative models are applied to the generation of high-quality samples, such as DCGAN [63], LAPGAN [64], and SAGAN [65]. Compared with the traditional generation models, these derivative models generate more diversity of samples and more real samples; these models are stable and are with faster convergence speed.  Table 5 shows four comparisons of GAN models in high-quality examples generation.

As early as 2014, the convolution neural network [66] has been used in the field of image super-resolution, but at that time, the convolution layer number was relatively small and the quality of the generated samples was poor. With the rapid development of deep learning algorithms. ResNet [67], DenseNet [68], and other multilayer neural network models have been applied to solve the problem of image super-resolution and achieved good results, but there are still some factors such as the difficulty of model convergence and the instability of model training. With the emergence of GANs, researchers have devoted themselves to developing new models to solve the problem of image super-resolution. DCGAN, WGAN, and LAPGAN have better performance in generating high-resolution images. Han Zhang et al. proposed the SAGAN model, which realized the task of image generation under attention mechanism and long-distance dependence. Compared with other GAN networks, SAGAN can generate high-resolution detail features by using spatial local points in low-resolution images. SAGAN can generate detailed information by using hints from all feature locations, and SAGAN adds spectral regularization to generation networks, which achieves better results than previous GAN-derived models in generating high-resolution images.

Video super-resolution (VSR) is a difficult problem in video processing. The main super-resolution algorithms based on deep learning are SRCNN, ESPCN, VESPCN, SRGAN, etc. These algorithms surpass traditional machine learning algorithms in dealing with image and video super-resolution problems. Alice Lucas et al. proposed a generation model based on the generative adversarial idea of the VSRResNet network model, combining with discriminator to guide the generator to generate new samples. Using traditional GAN models in video super-resolution will produce artifacts, so the original objective function needs to be regularized. Compared with the L2 regularization, the author uses the Charbonnier distance function to regularize feature space and pixel space. The function is as follows:

(20) $$\begin{array}{c} \gamma \left(x,y\right)=\sum_{i}\sum_{j}\sqrt{{\left(x_{i,j}-y_{i,j}\right)}^{2}+\varepsilon^{2}}, \end{array}$$

where *i* and *j* represent pixel coordinates and *ε* represents a very small constant. Using this function to provide regularization in the pixel space ensures that the super-resolution frame does not deviate greatly from the content of the corresponding real sample high-resolution frame. In the feature space, the deep feature is learned by discriminator to compare the reconstructed frames from the real sample. The feature space is composed of the features extracted from the third and fourth layers of the VGG network. The objective function is as follows:

(21) $$\begin{array}{c} \min_{\theta }\max_{\phi }L_{\text{total}}\left(\theta ,\phi \right)=\alpha \sum_{\left(x,Y\in T\right)}\gamma \left(VGG\left(x\right),\text{VGG}\left(G_{\theta }\left(Y\right)\right)\right)+\beta \left[E_{x}\left[\log D_{\phi }\left(x\right)\right]+E_{Y}\left[\log\left(1-D_{\phi }\left(G_{\theta }\left(Y\right)\right)\right)\right]\right]+\left(1-\alpha -\beta \right)\sum_{\left(x,Y\in T\right)}\gamma \left(x,G_{\theta }\left(Y\right)\right). \end{array}$$

A new model for video super-resolution, VSRResFeatGAN, is obtained. By using the PercepDist metric compared with the current video resolution model, the model has great advantages in quantitative and qualitative analysis.

### 4.3. Domain Transfer

Domain transfer is to transfer from one image domain to another, such as image style transfer and type conversion [69, 70]. Compared with traditional image translation methods, GANs are free from formal constraints and flexible to use. It can solve many different tasks at the same time. It provides a unified framework for different tasks through adversarial training. Several domain transfer models include Pix2pixGAN [71], CycleGAN [72], DiscoGAN [73], PAN, StarGAN [74], DTN [75], and Sim-GAN [76].  Table 6 shows five comparisons of GAN models in domain transfer tasks. Pix2pixGAN is an image style transfer model proposed by Isola et al. in 2017. Taking the satellite map generated by a plane map as an example, the generator uses a U-net structure. The input of the generator is a grid-like plane map, and the output is a false sample similar to the satellite map. The discriminator uses PatchGAN network architecture, which can effectively reduce the parameters of the discriminator and improve the training speed and efficiency. The discriminator inputs two pictures: one is the false sample image generated by the generator and the other is the real sample image of the satellite map. The discriminator determines which one is a true sample and which one is a false sample. In the adversarial training, the generated sample of the generator becomes more and more real; at the same time, the discriminator will not be able to distinguish which is the true sample. Finally, the network can generate images with a real sample style based on contour images. Pix2pixGAN is a supervised learning method relying on the CGAN model. It needs a one-to-one data set in training. The sample generated by Pix2pixGAN is more authentic with faster training speed.

Compared with Pix2pixGAN, CycleGAN is an unsupervised generation model, which has attracted much attention since it was proposed. The main idea is to add a new loss function: cyclic uniform loss function. To the traditional GAN objective function, the formula of the loss function is as follows:

(22) $$\begin{array}{c} L_{\text{cyc}}\left(G,F\right)=E_{\text{pdata}}\left(x\right)\left[{\left\|F\left(G\left(x\right)\right)-x\right\|}_{1}\right]+E_{\text{pdata}}\left(y\right)\left[\log\left(\left\|G\left(F\left(y\right)\right)-y\right\|\right)\right]. \end{array}$$

CycleGAN is trained in two steps. In the first step, it is the same as the Pix2pixGAN process. *Y* is the true sample set and *X* is the false sample set. After the first round of training, *X* under *Y* type will be generated. In the second step, the training process data set is just the opposite data set in the first step. *X* and *Y* will be exchanged in the training process. After the second round of training, *X* under *Y* type will be produced. In the two rounds of training, *X* and *Y* are not required to be the same object, as long as their potential models are the same. In error analysis, the model considers two training processes and uses reconstruction error to model. Specifically, the false sample *Y* generated in the first round is used as the input of the second generator to generate false sample *X*. The error distance between *X* and the false sample *X* needs to reduce. It is the same for *Y*; the weight parameters of two pairs of GAN network models can be optimized after error backpropagation. After such training, the purpose to transfer *Y* style to *X* can be achieved, where *X* and *Y* do not have the same limitations as Pix2pix GAN data sets. The objective function of the final CycleGAN model is as follows:

(23) $$\begin{array}{c} L\left(G,F,D_{X},D_{Y}\right)=L_{\text{GAN}}\left(G,D_{Y},X,Y\right)+L_{\text{GAN}}\left(G,D_{X},X,Y\right)+\lambda L_{\text{cyc}}\left(G,F\right). \end{array}$$

Although CycleGAN does not require a one-to-one data set, so the quality of the image generated by this model is not as good as Pix2pixGAN, but its application scenarios are rich and flexible.

**Figure 6:**
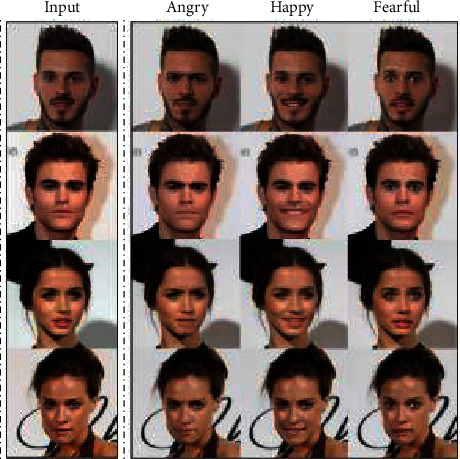
StarGAN generation image [71].

DiscoGAN is a generative model that can discover cross-domain relations. It generates cross-domain images through DTN, but its data set still requires a set of one-to-one images. PAN network has been introduced in Section 3.1. This network mainly adds perceptual adversarial loss to the traditional GAN model. The difference of the loss function is the diversity between the high-level features of true samples and false samples extracted from the discriminator. PAN can be used in more abundant scenes, which can carry out image dewatering and snow removal, label image generates street scene, satellite map generates a plane map, and contour map generates real images and image restoration, etc. StarGAN is a multidomain style transfer model. The network is based on CycleGAN, and a classifier is added to discriminator to classify domains. The image generated by the network is shown in Figure 6. SemGAN is a semantically consistent GAN model using a semantic segmentation algorithm. This model combines the loss function of traditional GAN with cyclic restriction loss function, which enhances semantic continuity and authenticity of generated results. As one of the most important applications of GAN, domain transfer has broad application prospects in the field of image style transfer and image translation. The future development mainly depends on semantic control to achieve image generation and also has great application in cross-domain and multidomain fields.

### 4.4. Image Restoration

Image restoration is a process of image reconstruction, which mainly includes restoring damaged images to the original image or generating a complete image from the local image. Dong et al. [77] proposed a remote sensing image restoration technology using the DCGAN network. This technology is different from the traditional method of restoring the surrounding information of a single image but restores the cloud-covered remote sensing image from a large number of historical image records. The experimental results show that using DCGAN to restore remote sensing image is better than traditional image restoration technology in quantitative and qualitative analysis. Image recognition in the underwater area has always been a difficult problem in the image recognition field. Due to the influence of turbulence, underwater images will be deformed, and the refraction of light will also cause geometric distortion. To improve the success rate of underwater image recognition, He et al. [78] proposed a method of underwater image distortion sequence restoration using a deep learning model. The author analyzed the advantages and disadvantages of traditional GANs. After that, DeblurGAN [79] is used as the network structure of image restoration. Firstly, the initial blurred image is obtained by using the time series of a distorted image, and the image is inputted into the generator. The generated false samples and real samples are distinguished by the discriminator. The discriminator uses Wasserstein distance to measure the distance between the true and false samples and uses it as the basis for optimizing the weighting parameters of the networks. DeblurGAN network structure is characterized by the use of residual blocks and jumps connection. Residual network [68] is easier to optimize the network structure. The use of jump connections improves the speed and efficiency of network training. DeblurGAN-v2 [80] is more efficient than DeblurGAN in deblurring. It is an end-to-end GAN for the dynamic deblurring of a single image. The GAN model based on relative condition constraints and the dual-mode discriminator is used, combining the pyramid feature network structure. It has faster deblurring than other networks.

In the field of face recognition, because of the pose or camera angle problem, it is impossible to obtain all facial features, so how to use local features to obtain the overall information is an urgent problem to be solved. Huang et al. [81] proposed a TP-GAN network model that combines global structural features and local details to generate complete images. It can synthesize frontal images from the image with different viewpoints, different illumination conditions, and different shooting positions. When one side of the face appears in the lens, the network model can also accurately recognize it.

Literature [82] proposes a new semantic image restoration technique, which uses existing image data to search for the nearest encoding of the damaged image in the hidden image manifold through context and prior loss and then puts the encoding into the generative model to infer the missing data part and generate the data information. The technology can predict the lost information with high accuracy and achieve the fidelity of pixel level in the case of less information loss, but in the case of more information loss, the accuracy of prediction results will be relatively low and cannot reach the high-quality image restoration level. In semantic image restoration, Pathak et al. [83], proposed an unsupervised feature learning algorithm, which generates the content of the missing area around the image according to the context encoding information. By using the comprehensive loss function composed of pixel reconstruction loss and perceptual adversarial loss to train model, the generated image is of higher quality than the supervised learning model. In the restoration of high-pixel missing images, PGGAN [84] combines the network structure G-GAN and pathGAN. By discriminator network to obtain global and local image information, PGGAN divides the discriminator network into two structures; one is to discriminate the authenticity of the samples, and the other is to evaluate the generated local details, combining the two structures' information and using reconstruction loss, generative adversarial loss, and connection loss as an objective function to optimize the model structure. The image generated by this model is superior to other generative models in visual effect and quality evaluation.

### 4.5. Application in AI Security

**Figure 7:**
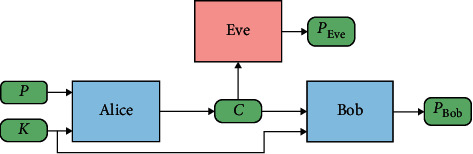
Encrypted communication system.

In addition to the above areas, GAN models also play a very important role in the field of security [85–87]. How to share data secretly in communication now has different encryption schemes, such as dynamic encryption. But they also have known shortcomings and insecurity problems. In 2016, Martin Abadi et al. [88] proposed a scheme of encryption and decryption in communication through GAN. Specifically, when the two sides communicate, they do not specify the specific cryptographic algorithm but implement the encryption and decryption process through end-to-end adversarial training. In the communication process shown in Figure 7, *P* represents plaintext, *K* represents a key, in the communication system of Alice and Bob, GAN ensures that Bob's loss in decryption process is reduced and Eve's loss in decryption process is increased, and Eve cannot decrypt the text through a neural network, thus ensuring the security of communication between Alice and Bob. This communication system realizes the confidentiality of a multiagent system through adversarial training.

Anomaly detection is one of the most important problems in a series of other fields, including medical imaging, manufacturing, and network security. The method of anomaly detection needs to model high-dimensional and complex data. The method of anomaly detection based on the GAN model proposed by Zenati [89] is effective. It learns the real data distribution through GAN, modeling the practical high-dimensional and complex data. When false data occurs, the discriminator in the GAN network can effectively detect anomalies. Using the GAN model to detect anomalies has more advantages than previous algorithms.

With the popularization and extension of AI technology in more and more fields, how to prevent the system from invading or attacking through AI is a serious problem at present. Feng Ji, the Executive Director of Nanjing International Institute of Artificial Intelligence [90], proposed a DeepConfuse technology, which adds a bounded perturbation to the training sample using a well-trained noise coder through hijacking the training process of neural networks. Adding a bounded perturbation makes the learning model acquired by training the perturbed samples have poor generalization ability of the test data; therefore, it realizes the function of “data poisoning”. In real scenarios, this technology will interfere with the normal learning of AI machines, which may not only make the AI model's generalization ability very poor but also make the purpose of the AI model fundamentally changed. For example, in automatic driving, AI recognizes obstacles as pathways or marks dangerous scenes as safety scenes. The purpose of DeepConfuse is to reveal the threat of AI intrusion or attack technology to system security, provide a feasible scheme for preventing related AI intrusion effectively, and play a guiding role in the research of AI security attack and defense.

## 5. Discussion and Conclusion

In this paper, we first review the latest research progress of computer vision, summarize the theoretical basis of GANs in detail, and elaborate on the challenges of GANs and the main advantages of GANs compared with traditional algorithms by combining its theoretical basis and practical use. The generative adversarial model has greatly promoted the rapid development of image processing field, with the continuous exploration of GAN models; they play an increasingly important role in other fields such as medicine, art, and security. For different fields and problems, more and more derivative models of GAN have come into people's vision. This paper lists some new derivative models and describes some recent research progress in computer vision, including data enhancement, high-quality sample generation, domain transfer, image restoration, and AI security.

To apply GAN models to different fields more rationally, we need to fully understand the advantages and disadvantages of GANs. In theory, GANs are trained based on the idea of minimax game, but they are difficult to achieve Nash equilibrium states in actual use and prone to gradient disappearance and model collapse. It is necessary to find more suitable objective functions or improve the traditional GAN network structures so that the models can guarantee the stability, convergence, and efficiency of the models in different fields to show the unique advantages of GANs. When combining other machine learning algorithms, GANs have broader application prospects, such as the following: (1) GANs combined with unsupervised learning algorithm have broad application prospects in forecasting problems; (2) GANs are used for target detection, through 3D modeling to understand the hidden law of things; (3) in AI medicine, GANs in the medical field can help doctors to carry out surgical treatment or disease detection; (4) GAN models are used to model large data, which provides new schemes for anomaly detection of data; (5) domain transfer has always been the most widely used area of GAN models, which will add a new color to the field of computer vision; (6) in AI security attack and defense, the generative model can replace human beings to carry out some intelligent operations. The security of autopilot, smart home, AI investment, and other high-risk areas needs to be considered. Therefore, GANs need to achieve a breakthrough in theory, solve some drawbacks of GAN models, and establish reasonable and accurate generative models through scientific and effective evaluation methods, taking into account the security and robustness when combined with different fields. In the future development of computer vision, the GANs will play more and more unique advantages.

## Figures and Tables

**Table 1:** Development of GANs.

Stages	Stage 1	Stage 2	Stage 3
Time	2014.06–2015.11	2015.11–2017.01	2017.1-today
GAN models	GAN- > DCGAN	DCGAN- > WGAN	WGAN- > today
Improvements	GAN is the beginning of generating the adversarial model	DCGAN uses many new methods to make the model more stable such as batchnorm, ReLU, and leaky ReLU	WGAN uses weight clipping solving the problem of gradient disappearance

**Table 2:** Loss function and derivative models for GANs.

GAN models	Loss function of discriminator	Loss function of generator
GAN [25]	*L*_*D*_=−*E*_*x*∼*p*_data__[log(*D*(*x*))] − *E*_*x*∼*p*_*z*__[log(1 − *D*(*G*(*z*)))]	*L*_*G*_=−*E*_*x*∼*p*_*z*__[log *D*(*G*(*z*))]

CGAN [8]	*L*_*D*_=−*E*_*x*∼*p*_data__[log(*D*(*x*, *c*))] − *E*_*x*∼*p*_*z*__[log(1 − *D*(*G*(*z*), *c*))]	*L*_*G*_=−*E*_*z*∼*p*_*z*__[log(1 − *D*(*G*(*z*), *c*))]

WGAN [39]	*L*_*D*_=−*E*_*x*∼*p*_data__[(*D*(*x*))] − *E*_*x*∼*p*_*z*__[(1 − *D*(*G*(*z*)))]	*L*_*G*_=−*E*_*x*∼*p*_*z*__[*D*(*G*(*z*))]

BEGAN [29]	*L*_*D*_=*E*_*x*∼*p*_data__[(*D*_AE_(*x*))]+*E*_*x*∼*p*_*z*__[max(0, *m* − *D*_AE_(*G*(*z*)))]	*L*_*G*_=−*E*_*x*∼*p*_*z*__[*D*_AE_(*G*(*z*))]

PAN [40]	*L*_*D*_=−*θL*_*D*_^GAN^+[*m* − ∑_*j*=1_^*F*^*λ*_*i*_*ℓ*_percep_^*D*,*j*^]	*L*_*G*_=*θL*_*G*_^GAN^+∑_*j*=1_^*F*^*λ*_*i*_*ℓ*_percep_^*D*,*j*^

InfoGAN [41]	*L*_*D*_=*L*_*D*_^GAN^ − *λL*_1_(*c*, *c*′)	*L*_*G*_=*L*_*G*_^GAN^ − *λL*_1_(*c*, *c*′)

LSGAN [42]	*L*_*D*_=−*E*_*x*∼*p*_data__[(*D*(*x*) − 1)^2^]	*L*_*G*_=−*E*_*x*∼*p*_*z*__[(*D*(*G*(*z*) − 1))^2^]

DRAGAN [43]	*L*_*D*_=*L*_*D*_^GAN^+*λE*_*z*∼*p*_*z*_+*n*(0, *c*)_[(‖∇*D*(*G*(*z*))‖ − 1)^2^]	*L*_*G*_=*E*_*z*∼*p*_*z*__[log(1 − *D*(*G*(*z*)))]

CWGAN [44]	*L*_*D*_=−*E*_*x*∼*p*_data__[*D*(*x*|*y*)]+*E*_*x*∼*p*_*z*__[*D*(*G*(*z*|*y*))]	*L*_*G*_=−*E*_*x*∼*p*_*z*__[*D*(*G*(*z*|*y*))]

AdaBalGAN [45]	*L*_*D*_=−∑_*i*=1_^*n*^log(*f*_*d*_(*M*_*r*_^*i*^))+∑_*i*=1_^*n*^log(*f*_*d*_(*M*_*s*_^*i*^))	*L*_*D*_=−∑_*i*=1_^*n*^[sign^*i*^ · log(*f*_*c*_(*f*_*g*_(*ε*^*i*^, sign^*i*^)))+(1 − sign^*i*^)log(1 − *f*_*c*_(*f*_*g*_(*ε*^*i*^, sign^*i*^)))] − ∑_*i*=1_^*n*^log(*f*_*d*_*M*_*s*_^*i*^)

FittingGAN [46]	*L*_*D*_=*L*_*D*_^CGAN^	*L*_*G*_=*L*_*G*_^CGAN^+*λE*_*x*,*y*_[‖*y* − *G*(*x*)‖_1_]

StackGAN [47]	*L*_*D*_=−*E*_(*I*, *t*)∼*p*_data__[log(*D*(*I*, *φ*_*t*_))] − *E*_*s*_0_∼*p*_*G*0_,*t*∼*p*_data__[log(1 − *D*(*G*(*s*_0_, *c*′), *φ*_*t*_))]	*L*_*G*_=*E*_*s*_0_∼*p*_*G*0_,*t*∼*p*_data__[log(1 − *D*(*G*(*s*_0_, *c*′), *φ*_*t*_))]+*λD*_KL_(*N*(*μ*(*φ*_*t*_), *E*(*φ*_*t*_))‖*N*(0,1))

**Table 3:** Comparisons of GAN models on the loss function.

GAN models	Improvements	Shortages	Applications
CGAN [8]	Through adding a conditional variable *c* to guide data generation and make the model faster to converge	The model is limited by data set and data set needs both tags and markeds	The model through semisupervised learning to generate a specified target

PAN [40]	The loss function was composed by the perceptual adversarial loss to train models	The model is a supervised model also needs the data set with both tags and markeds	The model can be applied to many image-to-image conversions

CWGAN [44]	The model is based on Wasserstein's condition which has a lower cost than traditional GANs	Model collapses and has a lack of diversity	The model can be applied to short data set's training

FittingGAN [46]	The model is based on the CGAN loss function but adds an L1 regularization	The model accuracy is not very high and has a lack of diversity	Be better than the image-to-image task, it can generate images different from the input image guide

**Table 4:** Comparisons of GAN models on the structure.

GAN models	Improvements	Shortages	Applications
MAD-GAN [49]	The model uses many generators and a discriminator to generate samples	Hard to convergence and lack of diversity	The model is applied to multivariate time series anomaly detection
InfoGAN [41]	The model's input is composed of *c* and *z*', through adding a classifier to predict code *c* that generates *x*	Complex and with a large number of params	The model is unsupervised and learns interpretable and disentangled representations on challenging datasets
ACGAN [4]	The model combines the advantages of CGAN and SGAN to generate samples	Semisupervised and the model is hard to converge in the small amount of data	Can generate high-quality samples and have diversity
StackGAN [50]	The model through two-stage training generating more realistic samples	Complicated and need more training time	The model can be applied according to text to generate images

**Table 5:** Comparisons of GAN models in high-quality examples generation.

GAN models	Improvements	Shortages	Applications
DCGAN [63]	The methods fraction-strided convolution, batchnorm, and ReLU make the model more stable and easy to converge	Model collapses and needs to adjust parameters in different conditions	Highest usage models in most scenarios
LAPGAN [64]	Laplacian and Gaussian pyramids in the up and down samples which make the model easy to approach and learn residuals	Supervision model	High-resolution images generation
SAGAN [65]	Using self-attention mechanism and two-timescale update rule, the model can generate realistic images	The attention mechanism is limited	Large-scale classification of conditional image generation tasks
VSRResFeat GAN [61]	Using GAN loss and Charbonnier distance in feature and pixel space	The noise in the estimated frames is redundant	Video super-resolution

**Table 6:** Comparisons of GAN models in domains transfer.

GAN models	Improvements	Shortages	Applications
Pix2pix [71]	Using U-NET network and PatchGAN architecture which make the model easy to converge and images are realistic	The model is a supervised model and also needs the data with both tags and markeds	Style transfer and other applications
CycleGAN [72]	Cycle loss, self-constraint, and two-step transformation. The model training does not need a large data set	The quality of generated images is lower than pix2pix	Most of the style conversion scenes
DiscoGAN [73]	Using two GAN models to discover cross-domain relationships reducing model collapse and improve image quality	Data sets must be one-to-one paired images	Most scenes in domain transfer
StarGAN [74]	Adding control information of a domain to understand the image which domain does it belongs to	Needs a large number of different data sets	Multidomain transfer
DTN [75]	Using several complex loss functions, generating appealing emoji. From a facial image	The generated images with low quality	Using real photos to generate cartoon images
